# Unveiling the bioinformatic genes and their involved regulatory mechanisms in type 2 diabetes combined with osteoarthritis

**DOI:** 10.3389/fimmu.2024.1353915

**Published:** 2024-08-08

**Authors:** Guangming Mao, Wenhao Xu, Lingli Wan, Hongpin Wang, Shutao Xu, Liangming Zhang, Shiyang Li, Jifa Zhang, Zhongming Lai, Yuping Lan, Jianhui Liu

**Affiliations:** ^1^ Department of Pharmacy, Panzhihua Central Hospital, Panzhihua, China; ^2^ Department of Pharmacy, Dali University, Dali, China; ^3^ College of Pharmacy and Bioengineering, Chongqing University of Technology, Chongqing, China

**Keywords:** type 2 diabetes mellitus, osteoarthritis, bioinformatics analysis, matrix metalloproteinase-9 (MMP9), angiopoietin-like 4 (ANGPTL4)

## Abstract

**Background:**

Type 2 Diabetes Mellitus (T2D) and Osteoarthritis (OA) are both prevalent diseases that significantly impact the health of patients. Increasing evidence suggests that there is a big correlation between T2D and OA, but the molecular mechanisms remain elusive. The aims of this study are to investigate the shared biomarkers and potential molecular mechanisms in T2D combined with OA.

**Methods:**

T2D and OA-related differentially expressed genes (DEGs) were identified via bioinformatic analysis on Gene Expression Omnibus (GEO) datasets GSE26168 and GSE114007 respectively. Subsequently, extensive target prediction and network analysis were finished with Gene Ontology (GO), protein-protein interaction (PPI), and pathway enrichment with DEGs. The transcription factors (TFs) and miRNAs coupled in co-expressed DEGs involved in T2D and OA were predicted as well. The key genes expressed both in the clinical tissues of T2D and OA were detected with western blot and qRT-PCR assay. Finally, the most promising candidate compounds were predicted with the Drug-Gene Interaction Database (DGIdb) and molecular docking.

**Results:**

In this study, 209 shared DEGs between T2D and OA were identified. Functional analysis disclosed that these DEGs are predominantly related to ossification, regulation of leukocyte migration, extracellular matrix (ECM) structural constituents, PI3K/AKT, and Wnt signaling pathways. Further analysis via Protein-Protein Interaction (PPI) analysis and validation with external datasets emphasized MMP9 and ANGPTL4 as crucial genes in both T2D and OA. Our findings were validated through qRT-PCR and Western blot analyses, which indicated high expression levels of these pivotal genes in T2D, OA, and T2D combined with OA cases. Additionally, the analysis of Transcription Factors (TFs)-miRNA interactions identified 7 TFs and one miRNA that jointly regulate these important genes. The Receiver Operating characteristic (ROC) analysis demonstrated the significant diagnostic potential of MMP9 and ANGPTL4.Moreover, we identified raloxifene, ezetimibe, and S-3304 as promising agents for patients with both T2D and OA.

**Conclusion:**

This study uncovers the shared signaling pathways, biomarkers, potential therapeutics, and diagnostic models for individuals suffering from both T2D and OA. These findings not only present novel perspectives on the complex interplay between T2D and OA but also hold significant promise for improving the clinical management and prognosis of patients with this concurrent condition.

## Introduction

Diabetes mellitus is a prevalent chronic metabolic disorder, mainly manifested as type 2 diabetes mellitus (T2D), which accounts for more than 90% of adult cases of diabetes (1). According to reports, approximately 537 million adults aged 20-79 were diagnosed with diabetes globally in 2021, accounting for 10.5% of the world’s adult population (2, 3). Projections indicate that this number will increase to 643 million by 2030 and further to 783 million by 2045. Additionally, there has been a rise in the incidence of T2D among adolescents under 18 years old (4). The pathogenesis of the disease involves insulin resistance, secretion defects, genetics, and other factors. Research has also linked T2D with inflammatory factors such as C-reactive protein, IL-6, plasminogen activator inhibitor 1, TNF-α, and chemokines, noting that adipokines from adipose tissue may aggravate insulin resistance-associated inflammation (5–7).

Osteoarthritis (OA) is a chronic condition that primarily affects weight-bearing joints such as the knees and hips, potentially involving the entire joint tissue and impeding movement. It significantly deteriorates the quality of life for patients and has an adverse impact on mental health, including depression, sleep disorders, and suicidal tendencies (8, 9). As a results, it imposes a considerable economic burden on individuals and society. In 2019, there were 528 million reported cases of OA globally, with a prevalence rate of 6.8% (10). The fundamental pathological change in OA is the deformation and destruction of articular cartilage. Its complex etiology involves biomechanical factors, pro-inflammatory mediators, obesity, proteases, aging, immunity, genetics, and hormonal influences (11–13).

From 2019 to 2022, the department of orthopedics at Panzhihua Central Hospital treated 458 inpatients with T2D combined with OA, representing 8.25% of OA patients. Recent case reports and studies suggest a significant increase in metabolic syndrome incidence among OA patients, with T2D potentially being an independent risk factor for OA development (14). Epidemiological surveys indicate that T2D patients are more prone to OA, with a higher OA risk compared to controls (15, 16). An animal study using streptozotocin-induced diabetes in rats showed resistance to insulin-like growth factors in cartilage and significant synovial and collagen changes after 70 days, highlighting the link between diabetes and OA (17). The concept of diabetic osteoarthritis (DO) has emerged, with increasing research on its common pathogenesis. However, the precise mechanisms remain unclear, and specific effective treatments for DO are still lacking. Further investigation into the pathogenesis and identification of key targets is crucial for developing therapeutic drugs or methods, significantly impacting disease prevention and treatment.

**Figure 1:**
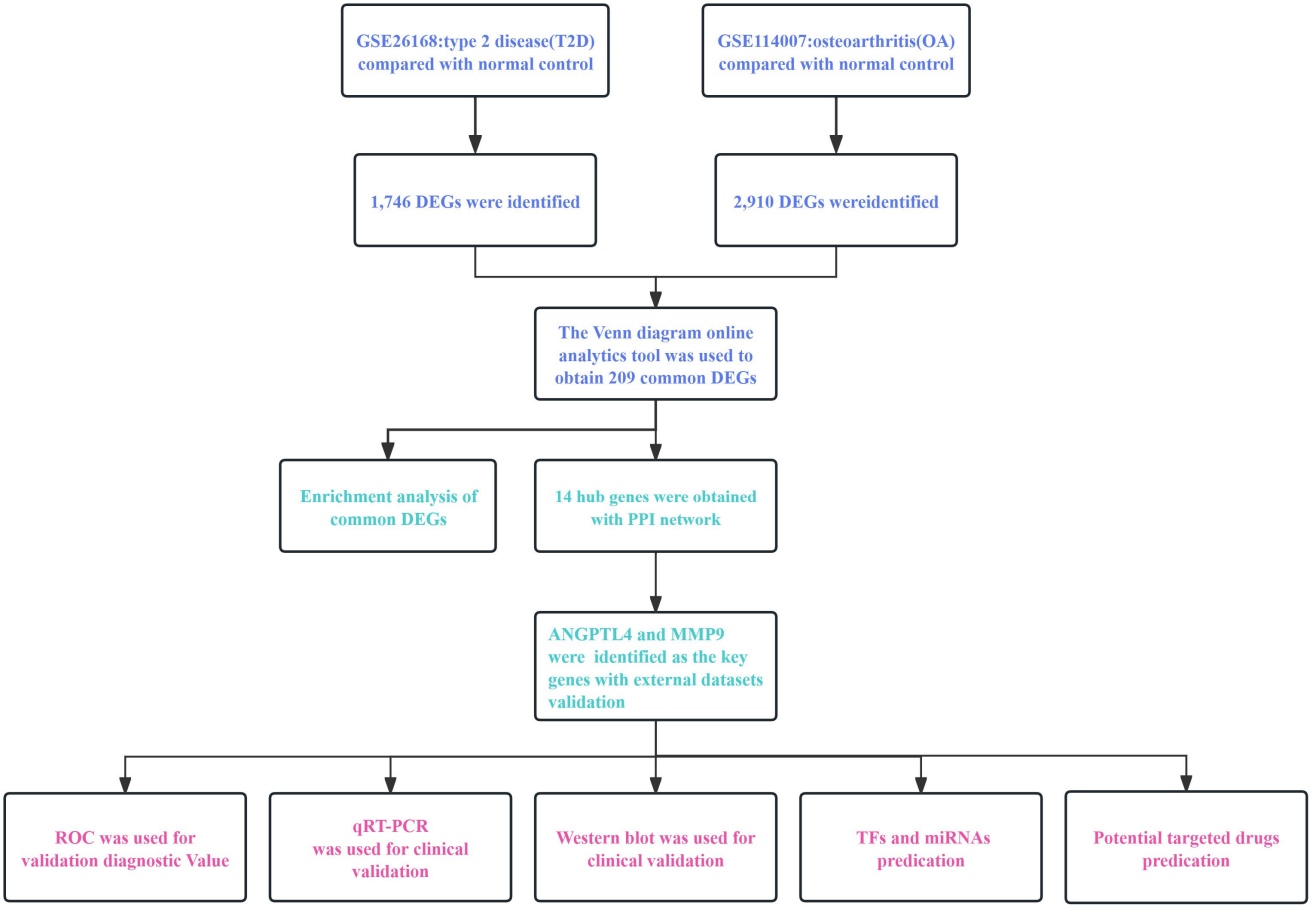
The workflow diagram of this research. T2D, Type 2 Diabetes Mellitus. OA, Osteoarthritis. PPI, protein-protein interaction. DEGs, differentially expressed genes.

In this study, we constructed detailed process flow diagrams to illustrate the critical steps involved (**Figure 1**). We utilized bioinformatics to identify common differentially expressed genes (DEGs) between T2D and OA. We conducted Protein-Protein Interaction (PPI) network analysis and visualization for the DEGs, and validated our findings with external datasets. Our results identified angiopoietin-like 4 (ANGPTL4) and matrix metalloproteinase 9 (MMP9) as pivotal genes in both T2D and OA. Additionally, we assessed the accuracy of these genes as biomarkers for disease prediction and diagnosis using receiver operating characteristic curve (ROC). Furthermore, we explored potential drugs targeting these key genes through molecular docking.

## Materials and methods

### Data source

We obtained our data from the Gene Expression Omnibus (GEO) database (http://www.ncbi.nlm.nih.gov/geo). Using “type 2 diabetes mellitus” and “osteoarthritis” as search keywords, we selected the GSE26168 and GSE114007 datasets for our screening analysis. The selected dataset which contains more samples and comprehensive clinical information has good quality and meets the requirements of research analysis. The GSE26168 dataset consists of gene expression data from the blood tissue of nine patients with type 2 diabetes mellitus (T2D) and eight healthy controls. The GSE114007 dataset includes gene expression information from the cartilage tissue of 20 patients with osteoarthritis (OA) and 18 normal controls. To validate our findings, we also retrieved the GSE20966 (T2D) and GSE51588 (OA) datasets from the GEO database.

### Screening of DEGs

We utilized RStudio software (version 4.2.1) and the “Limma” R package to process the datasets and identify DEGs between the disease and control groups (18, 19). To ensure accuracy, we excluded probe sets without corresponding gene symbols and calculated averages for genes represented by multiple probe sets. Our significance threshold was set at p<0.05 and |logFC|>1. Additionally, we used the Venn diagram online analysis tool (http://bioinformatics.psb.ugent.be/webtools/Venn/) to identify common DEGs between T2D and OA datasets.

### Enrichment analysis of common DEGs

To analyze the functional characteristics of genes shared by T2D and OA, we utilized RStudio software for Gene Ontology (GO) and Kyoto Encyclopedia of Genes and Genomes (KEGG) enrichment analysis, using the “cluster Profiler” R package. GO is an internationally recognized gene functional classification system that provides a comprehensive and regularly updated vocabulary, as well as well-defined concepts for identifying genes and their products (20). It consists of three ontologies: molecular function, cellular component, and biological process. GO enrichment analysis allows for the identification of significantly enriched GO terms compared to the genome background, and associates DEGs with specific biological functions. KEGG, a principal public database for pathway-related information, enables the identification of pathways that are significantly enriched in DEGs compared to the genome-wide background (21).

### PPI network

Using the STRING online tool (http://string-db.org), we explored the interactions among common DEGs and constructed a Protein-Protein Interaction (PPI) network with extensive regulatory links (22). The STRING database facilitates the search for relationships between proteins, encompassing direct binding relationships and interconnected upstream and downstream regulatory pathways. Interactions with a confidence value above 0.4 were deemed statistically significant, with all other parameters set to default. We employed Cytoscape software (version 3.8.2) for PPI network analysis and visualization and utilized the Cytoscape plug-in, Molecular Complex Detection (MCODE), for identifying primary functional modules (setting k-core=2, degree cutoff=2, max depth=100, node score cutoff=0.2) (23, 24). Key genes were identified using Cytoscape’s CytoHubba plugin (25), and an upset diagram was used to filter and determine shared key genes. Additionally, we established a co-expression network for these key genes using GeneMANIA (http://www.genemania.org/), a tool renowned for uncovering the internal relationships within gene sets (26).

### Identification of key genes

We utilized the GSE20966 (T2D) and GSE51588 (OA) datasets to validate the mRNA expression of key genes. This process aimed to definitively identify the key genes for T2D and OA. The GSE20966 dataset includes samples from 10 T2D patients and ten controls, while the GSE51588 dataset comprises 40 OA patient samples and ten control samples.

### qRT-PCR

Quantitative real-time polymerase chain reaction (qRT-PCR) was employed to assess the mRNA expression of key genes in T2D (n=8, peripheral blood), OA (n=7, articular cartilage), T2D combined with OA (T2D+OA) samples (n=4,articular cartilage), and normal controls (n=9,peripheral blood). The inclusion and exclusion criteria for the clinical study were listed in **Supplementary Table 1**. Clinical tissue samples were acquired from Panzhihua Central Hospital. For the study, we used TRIpure Total RNA Extraction Reagent (EP013, ELK Biotechnology) to extract RNA from the samples. The isolated total RNA was reverse-transcribed into complementary DNA (cDNA) using the EntiLink™ 1st Strand cDNA Synthesis Super Mix (EQ031, ELK Biotechnology). qRT-PCR was performed with the QuantStudio 6 Flex System PCR instrument (Life Technologies) and the EnTurbo™ SYBR Green PCR SuperMix kit. Each sample was prepared in triplicate wells. β-actin served as the internal reference gene, and the 2-ΔΔCt method was used for calculating mRNA fold changes and normalizing the data across all samples. The primer sequences for PCR are listed in **Supplementary Table 2**.

### Western blot

For protein extraction, we initially rinsed 3 samples each of T2D peripheral blood, OA articular cartilage, T2D+OA articular cartilage, and peripheral blood from healthy controls with pre-chilled PBS buffer (ASPEN, AS1025). This process was repeated 2-3 times. Subsequently, RIPA total protein lysate (ASPEN, AS1004) was added in volume 10-20 times that of the tissue samples. The mixture was then centrifuged at 12,000 rpm at 4°C for 5 minutes, and the supernatant was collected. Protein concentration in the samples was determined using the BCA Protein Concentration Assay Kit (ASPEN, AS1086). For western blot (WB), SDS-PAGE electrophoresis was performed. An appropriate amount of 5×protein loading buffer (ASPEN, AS1011) was added to the samples, which were then incubated in a boiling water bath at 95-100°C for 5 minutes. Electrophoresis was conducted at 80V for the stacking gel and 120V for the separation gel. A PVDF membrane (Millipore, IPVH00010) was activated with methanol for 3 minutes before use. The transfer of proteins onto the membrane was carried out at a constant flow of 300mA.The membranes were immunoblotted with the diluted primary antibodies (ASPEN, AS1061) for overnight at 4°C. The blots were washed thrice with TBST. Afterward, the diluted secondary antibody was incubated at room temperature for 30 minutes. Excess antibody was washed off with TBST for 4 times. Immunoreactivity was detected using ECL western blot reagent. Finally, the signal bands were quantified by densitometry analysis using the AlphaEaseFC software processing system after scanning the blotted membrane.

### Diagnostic value of key genes

To ascertain the diagnostic value of pivotal genes in OA, we utilized external datasets GSE20966 (T2D) and GSE51588 (OA) to validate clinical diagnosis prediction models. Employing RStudio software (version 4.2.1), we used the pROC package to generate Receiver Operating Characteristic (ROC) curves. Additionally, logistic regression analysis was conducted to evaluate the role of key genes in distinguishing between T2D, OA, and healthy individuals. This analysis facilitated the establishment of a clinical diagnosis prediction model based on the identified key genes.

### Prediction of transcription factors

Transcription factors (TFs) are proteins that bind to specific DNA sequences, forming complex regulatory systems to control gene expression. We utilized Network Analyst 3.0 (https://www.networkanalyst.ca/) to examine the interactions between key genes and transcription factors in T2D and OA (27). This analysis aimed to evaluate the impact of TFs on the expression and functional pathways of key genes. The transcription factor and gene target data were sourced from the ENCODE ChIP-seq database (28). We applied a peak intensity signal threshold of <500 and a regulatory potential score of <1 point, as predicted using the BETA negative algorithm. Furthermore, we employed Cytoscape to visually represent the TFs-mRNA regulatory network.

### Prediction of microRNAs

MicroRNAs (miRNAs) are endogenous, short, non-coding RNAs that play a crucial role in inhibiting or degradating target mRNAs. In order to better understand how gene expression is affected in different physiological and disease contexts, we analyzed the regulatory network between miRNAs and mRNAs (29). To do this, we utilized miRTarBase (version 8.0), a database specifically designed for predicting miRNA binding sites, to identify potential interactions between key genes and miRNAs. This involved conducting a cross-analysis of mRNA-miRNA binding and constructing a competing endogenous RNA (ceRNA) network. The results of this analysis were then visualized using Cytoscape software (version 3.8.0).

### Prediction of potential compounds

The Drug-Gene Interaction Database (DGIdb) (http://www.dgidb.org) is a comprehensive resource that provides information on interactions between drugs and genes (30). In this study, we utilized DGIdb to identify potential drugs that target pivotal genes, supplemented by candidate compounds sourced from existing literature for treating T2D and OA. We downloaded protein and ligand files for MMP9 and ANGPTL4 from the RCSB Protein Data Bank (https://www.rcsb.org/) in “PDB”. The PDB format focuses on storing 3D structural data of biological macromolecules such as proteins and nucleic acids, and contains detailed information of protein-ligand binding sites. It is more suitable for the screening of predictive gene drugs. The SDF format is mainly used to store and exchange detailed molecular structure information, including 2D or 3D coordinates of molecules, atomic types, bond information. It is suitable for occasions that require batch processing of large amounts of molecular data, such as virtual screening and compound library management (31). Therefore, after establishing the corresponding coordinates between the proteins and ligands, we converted the protein files to the “PDBQT” format. The “SDF” format ligands of candidate compounds were also converted into “PDBQT” format using the PubChem database (https://pubchem.ncbi.nlm.nih.gov/) (32). Molecular docking was performed using AutoDock (Vina 1.2.2), which calculated the binding affinity between receptors and ligands. A binding energy lower than −5.0 kcal/mol typically indicates strong binding activity to the target protein. We analyzed the docking results with the Protein-Ligand Interaction Profiler (https://plip-tool.biotec.tu-dresden.de/plip-web/plip/index) and visualized them using PyMOL (version 2.0). This allows us to demonstrate the ligand-receptor binding process through hydrogen bonds and amino acid residues.

### Statistical analysis

All data were processed and analyzed using the GraphPad Prism software 8.0 (GraphPad Software, La Jolla, CA, USA), and results were expressed as mean ± standard deviation (SD). Significant differences between the groups were evaluated by unpaired student’s *t*-test. Unidirectional Analysis of Variance (ANOVA) with Tukey’s multiple comparison tests was utilized where appropriate in cases involving multiple groups. A p-value of less than 0.05 was regarded as significant.

## Results

### Screening of DEGs in T2D and OA

**Figure 2:**
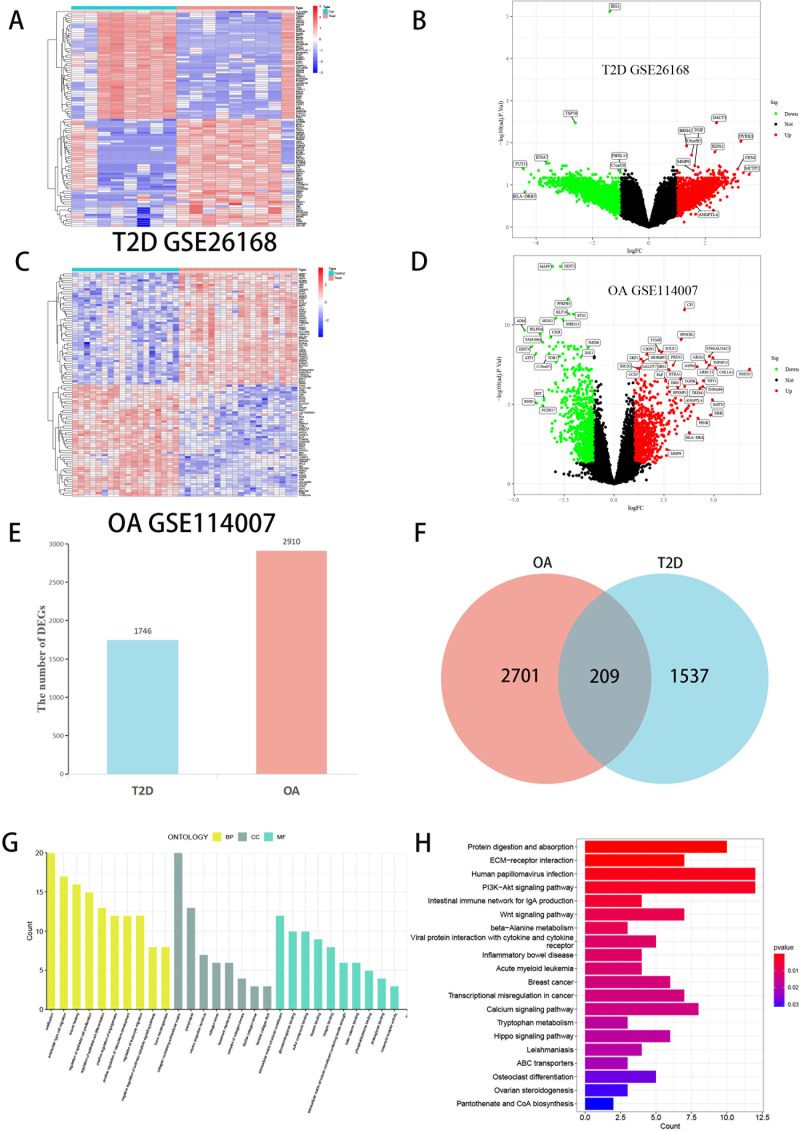
Visualization of DEGs screening of T2D and OA. **(A)** Heatmap of T2D datasets (GSE26168). **(B)** The volcano plot of DEGs in T2D datasets. **(C)** Heatmap of OA datasets (GSE114007). **(D)** The volcano plot of DEGs in OA datasets. Red represents upregulated genes, Green represents downregulated genes, and black represents genes with no difference. **(E)** Comparing the number of DEGs between T2D and OA. **(F)** Venn diagram showing the overlap of DEGs between T2D and OA. **(G)** The bar graphs of GO enrichment analysis. **(H)** The bar graphs of KEGG enrichment analysis. DEGs, differentially expressed genes. T2D, Type 2 Diabetes Mellitus. OA, Osteoarthritis.

We analyzed tissue samples from GEO, performing data on the datasets for T2D and OA. In the T2D dataset (GSE26168), we identified 1,746 DEGs. Similarly, in the OA dataset (GSE114007), we identified 2,910 DEGs. These DEGs for both T2D and OA were visually represented through volcano plots and heat maps (**Figures 2A–D**). A Venn diagram was utilized to highlight the overlap between the two datasets, revealing 209 common DEGs (**Figures 2E, F**). The results underscore the presence of many common genes shared between T2D and OA.

### Functional analysis of DEGs

GO and KEGG enrichment analyses were conducted on the 209 common DEGs. The GO analysis (**Figure 2G**) revealed significant gene enrichment in various categories. In Biological Processes (BP), the most enriched pathways included ossification, ameboidal-type cell migration, wound healing, and regulation of epithelial cell proliferation. Within the Cellular Components (CC) category, collagen-containing extracellular matrix, presynapse, and neuron projection terminus were most prominent. Regarding Molecular Functions (MF), the top enriched functions were extracellular matrix structural constituent (ECM), glycosaminoglycan binding, and sulfur compound binding. KEGG analysis (**Figure 2H**) indicated substantial enrichment in pathways such as ECM-receptor interaction, PI3K-Akt signaling pathway, Wnt signaling pathway, intestinal immune network for IgA production, and protein digestion and absorption. These results strongly suggest that the common DEGs between T2D and OA play crucial roles in regulating chemokines, cytokines, and inflammatory responses. These genes are implicated in cellular processes like production, metabolism, and apoptosis and are significantly associated with the onset and progression of T2D and OA.

### PPI network construction of hub genes

**Figure 3:**
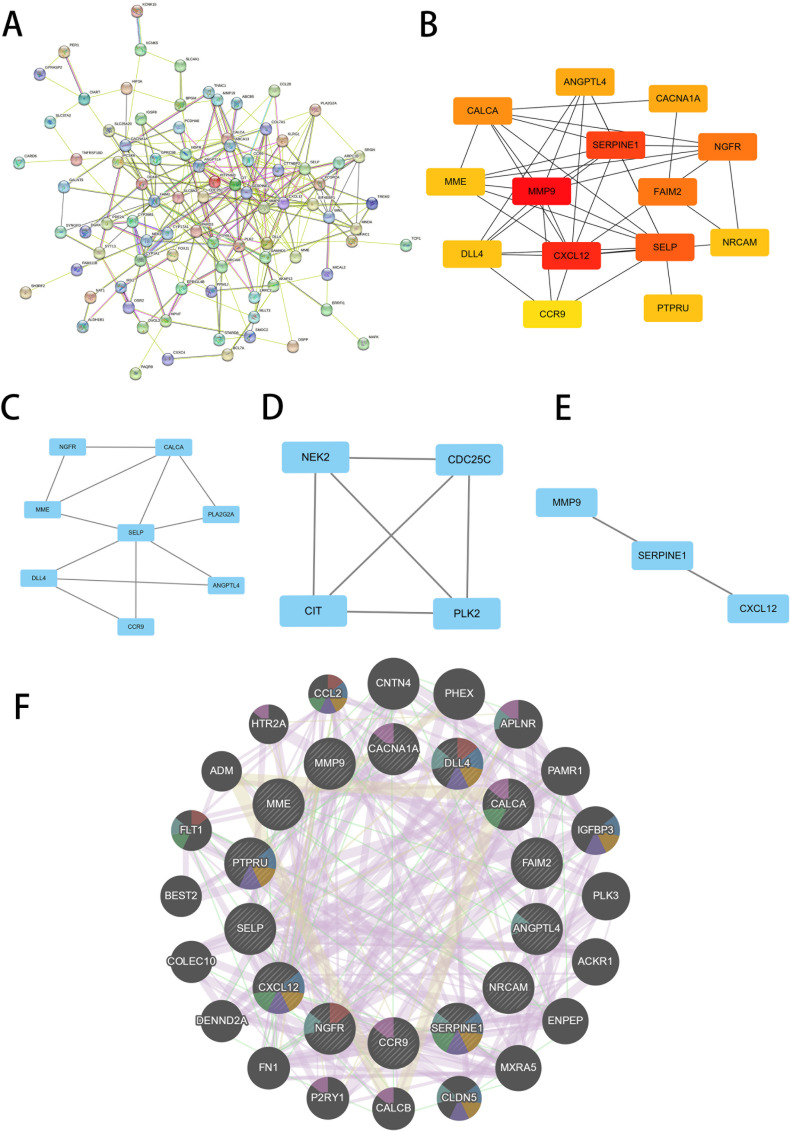
Screening of hub genes. **(A)** PPI network of 209 common DEGs between T2D and OA. **(B)** PPI network of top14 hub genes screened by the degree method using cytoHubba. A higher ranking is represented by a redder color. **(C–E)** Three significant gene clustering modules. **(F)** 14 hub genes and their co-expression genes were analyzed via GeneMANIA. PPI, protein-protein interaction. T2D, Type 2 Diabetes Mellitus. OA, Osteoarthritis.

We conducted a comprehensive analysis of the DEGs shared by T2D and OA using the STRING online network tool and Cytoscape software to elucidate PPI. A universal PPI network was constructed with a minimum interaction score of 0.4, consisting of 207 nodes and 226 edges (**Figure 3A**). This complex PPI network showed that 96.86% of the genes had co-expression and 2.05% shared protein domains, and 1.08% had interaction responses. In this network, nodes represent proteins, and edges represent their interactions. The size and color intensity of a node indicates its degree value, while the thickness of an edge reflects the strength of the relationship between the proteins. Using the Cytoscape plug-in MCODE, we identified the most significant gene module (**Figure 3B**). Furthermore, we used the Cytoscape’s cytoHubba plug-in to determine the top 14 hub genes, which included MMP9, NGFR, CXCL12, CACNA1A, SERPINE1, CCR9, CALCA, NRCAM, MME, ANGPTL4, PTPRU, DLL4, SELP, and FAIM2 (**Figures 3C–E**). To further explore the functions of these hub genes, we analyzed their co-expression network and related functions using the GeneMANIA database (**Figure 3F**).

### Identification of key genes

**Figure 4:**
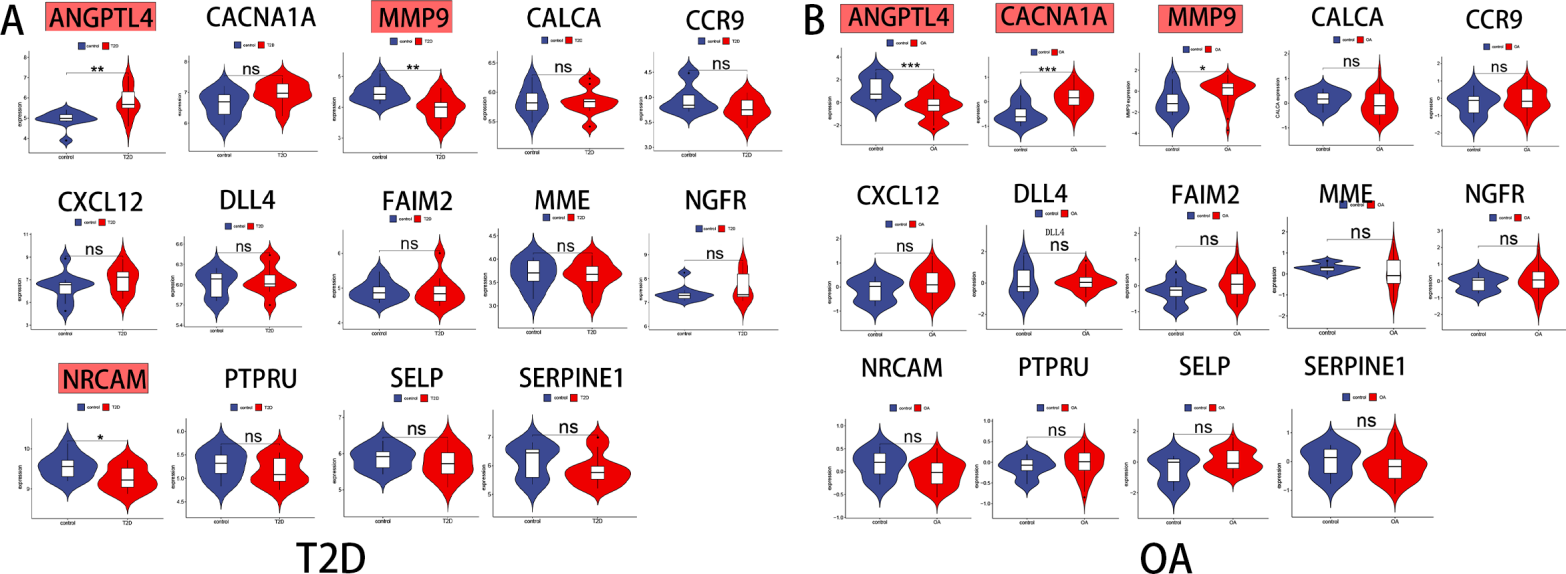
Identification of the key genes expression in external datasets. **(A)** ANGPTL4,MMP9 and NRCAM were statistically significant in T2D expression profifile (GSE20966). **(B)** ANGPTL4, MMP9 and CACNA1A were statistically significant in OA expression profifile (GSE51588). NS, not significant.;*, p < 0.05; **, p <0.01; ***, p< 0.001. T2D, Type 2 Diabetes Mellitus. OA, Osteoarthritis.

To validate the mRNA expression levels of the 14 identified hub genes, we utilized an additional T2D dataset and an OA dataset. The analysis of these datasets revealed significant expression differences in the hub genes. In the T2D external dataset (GSE20966), three hub genes-ANGPTL4, MMP9, and NRCAM-showed substantial differential expression compared to the normal control (**Figure 4A**). Similarly, in the OA external dataset (GSE51588), three hub gene-ANGPTL4, MMP9, and CACNA1A-exhibited significant expression differences (**Figure 4B**). By intersecting the results from both datasets, ANGPTL4 and MMP9 were conclusively identified as the pivotal genes shared between T2D and OA.

### Expression of the key genes in clinical samples

**Figure 5:**
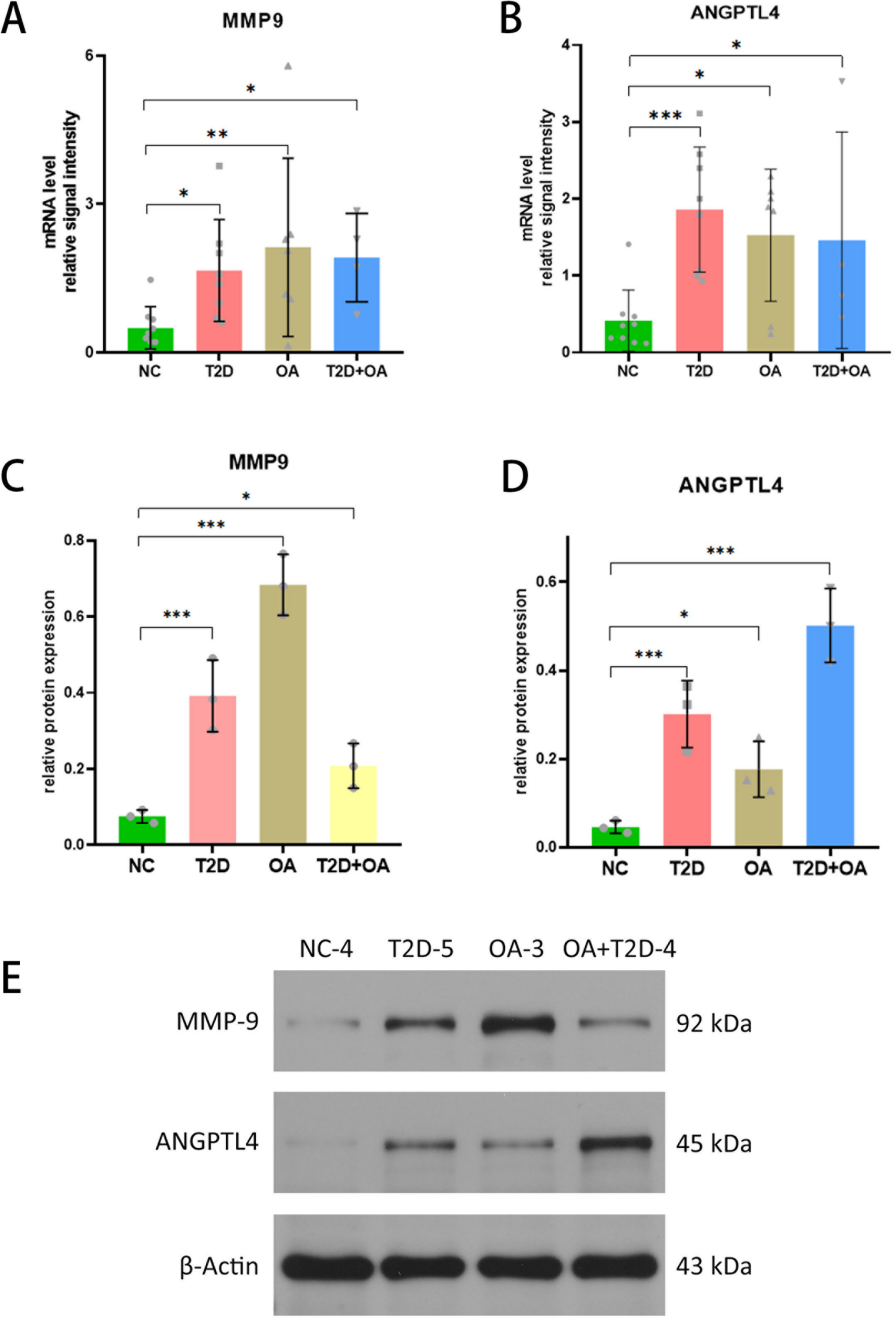
Validation of the key genes (*ANGPTL4* and *MMP9*). **(A)** MMP9 expression levels in tissue samples of NC, T2D, OA and T2D+OA. **(B)** ANGPTL4 expression levels in tissue samples of NC, T2D, OA and T2D+OA. **(C–E)** Western blot was used to determine the protein expression levels of *ANGPTL4* and *MMP9* in tissue samples of NC,T2D, OA and T2D + OA. *, p < 0.05; **, p <0.01; ***, p< 0.001. NC,Normal control. T2D+OA, T2D combined with OA. T2D, Type 2 Diabetes Mellitus. OA, Osteoarthritis. NC,normal control.

Firstly, we statistically analyzed the clinical data of the tested samples and established a baseline table of clinical information (**Table 1**). In the limited clinical cases, we can find that patients in the T2D + OA group had the highest blood glucose and Glycated hemoglobin A1c (GHbA1c), and 5 patients who had poor long-term blood glucose control in T2D group were rated grade 1 or 2 for Kellgren-Lawrence (KL). Four patients in OA group were in the abnormal range of blood glucose. There were no significant differences in age, sex and bone mineral density among the groups. It suggested that hyperglycemia may promote the development of osteoarthritis. Secondly, As illustrated in the figure, the test results indicated significant differences in the mRNA expression levels of these genes among the different groups. Specifically, MMP9 expression was significantly higher in T2D patients (p=0.046), OA patients (P=0.009), and T2D + OA patients (p=0.048) compared to healthy individuals (**Figure 5A**). Similarly, ANGPTL4 showed significantly elevated mRNA expression levels in T2D patients (P=0.001), OA patients (p=0.013), and T2D + OA patients (p=0.045) relative to healthy controls (**Figure 5B**).

**Table 1:** Basic information of the clinical samples.

Clinical indicators	T2D(n=8)	T2D+OA(n=4)	OA(n=7)	Normal(n=9)
Age (year), Mean(SD)	57.63(7.33)	57.25(6.80)	63.14(5.11)	59.00(4.74)
Gender (male/female)	5(3)	2(2)	4(3)	4(5)
IBM (kg/m²), Mean(SD)	25.05(2.61)	24.98(2.36)	27.72(2.52)	24.88(2.69)
Fasting glucose (mmol/l), Mean(SD)	9.37(2.48)	13.34(2.25)	5.83(1.09)	5.19(0.53)
GHbA1c(%), Mean(SD)	6.94(1.11)	11.31(1.24)	6.08(1.15)	4.44(1.04)
Kellgren-Lawrence grade (0/1/2/3/4)	3(4/1/0/0)	0(0/0/1/3)	0(0/0/2/5)	8(1/0/0/0)
Bone mass grade (1/2/3/4)	7(1/0/0)	4(0/0/0)	5(2/0/0)	8(1/0/0)
MMP9 expression levels, Mean(SD)	1.66(1.03)	1.92(0.90)	2.13(1.80)	0.50(0.43)
ANGPLT4 expression levels, Mean(SD)	1.86(0.81)	1.46(1.41)	1.53(0.86)	0.41(0.40)

Combined with the clinical data above, we can find that blood glucose was also higher in T2D and T2D + OA patients with higher MMP9 and ANGPTL4 expression. In T2D patients who had not been diagnosed with OA, higher expressions of MMP9 and ANGPTL4 were associated with higher KL grades. It suggests that the expression of MMP9 and ANGPTL4 may be positively correlated with blood glucose and the severity of osteoarthritis.The details on clinical data above can be found in the **Supplementary Table 3**.

### Protein analysis of hub genes

We utilized western blot analysis to evaluate the protein levels of ANGPTL4 and MMP9 in tissue samples from T2D patients, OA patients, T2D+OA patients, and healthy controls. The results showed a significant increase in MMP9 protein in T2D patients (5.57 times higher, p < 0.001), OA patients (9.71 times higher, p < 0.001), and T2D+OA patients (3times higher, p = 0.046), when compared to healthy controls (**Figures 5C, E**). Similarly, ANGPTL4 protein level was elevated in in T2D patients (6 times higher, p=0.001), OA patients (3.6 times higher, p=0.036), and T2D+OA patients (10 times higher, p < 0.001) compared to the healthy group (**Figures 5D, E**). The original western blot gel was found in the **Supplementary Figure 1**.

### Evaluation of key genes in T2D and OA

**Figure 6:**
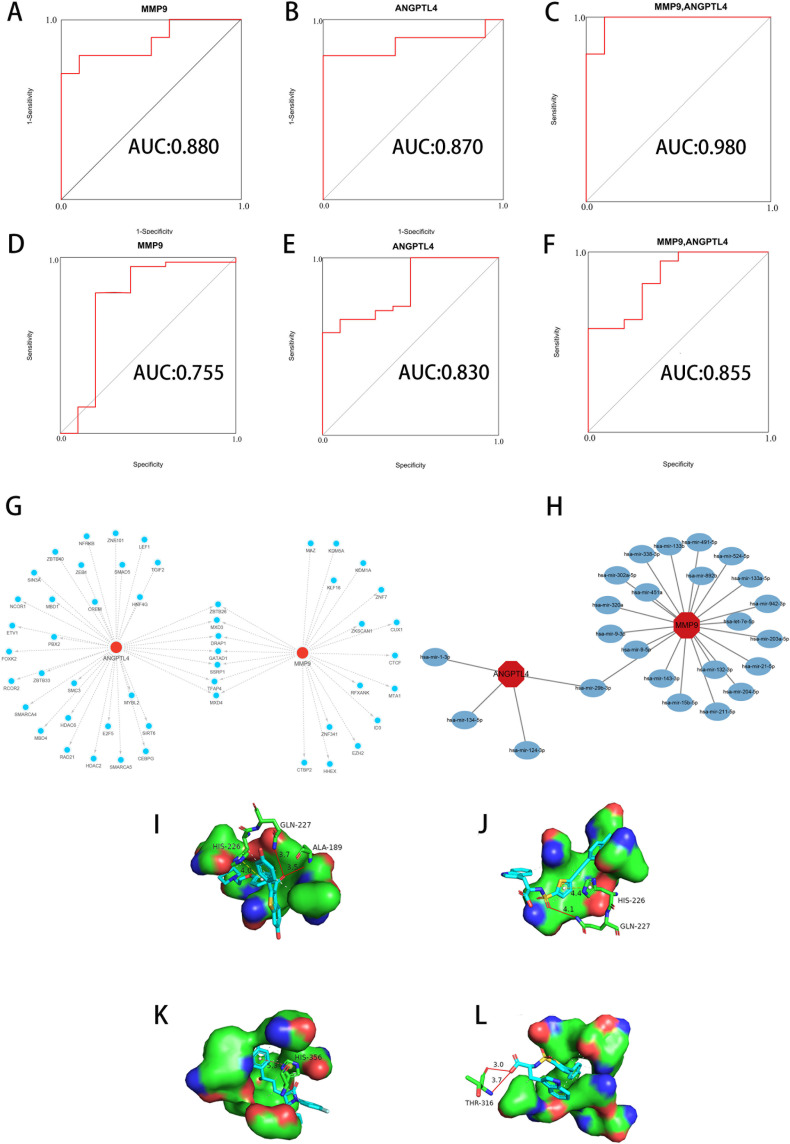
Diagnostic value, regulators, and potential drug analysis of key genes. **(A, B)** ROC of ANGPTL4 and MMP9 in the T2D dataset. **(C)** Validation of the integrated diagnostic model in the T2D dataset. **(D, E)** ROC of ANGPTL4 and MMP9 in the OA dataset. **(F)** Validation of the integrated diagnostic model in the OA dataset. These findings indicated these key genes have excellent diagnostic efficiency in T2D and OA. **(G)** TFs-gene interaction network based on key genes. The nodes in red color indicated key genes, and nodes in blue color indicated TF genes. **(H)** miRNAs-gene coregulatory network based on key genes. The nodes in red color indicated key genes, and nodes in blue color represent miRNAs. **(I)** Molecular docking conformation of Raloxifene interaction with MMP9. **(J)** Molecular docking conformation of S-3304 interaction with MMP9. **(K)** Molecular docking conformation of Ezetimibe interaction with ANGPTL4. **(L)** Molecular docking conformation of S-3304 interaction with ANGPTL4. ROC, Receiver Operating Characteristic. TFs, Transcription factors. miRNAs, MicroRNAs. T2D, Type 2 Diabetes Mellitus. OA, Osteoarthritis.

The method focused on assessing the area under curve (AUC) values to determine the sensitivity and specificity of these genes in diagnosing T2D and OA. In the T2D dataset, both ANGPTL4 and MMP9 had AUC values greater than 0.8 (**Figures 6A, B**), indicating a high level of diagnostic accuracy. Similarly, in the OA dataset, both genes had AUC values exceeding 0.75 (**Figures 6D, E**), confirming their strong diagnostic value for OA. Furthermore, we integrated ANGPTL4 and MMP9 with multiple markers to enhance prognosis prediction and developed a multi-marker diagnostic model using logistic regression analysis. The ROC results demonstrated that this multi-marker model effectively predicted the diagnosis of T2D (AUC = 0.980) and OA (AUC = 0.855) (**Figures 6C, F**), highlighting its potential clinical utility.

### Analysis of TFs-miRNAs regulatory network

The results in the construction of an interaction network between key genes and TFs (**Figure 6G**). The network consists of 50 nodes and 57 edges. Notably, ANGPTL4 is influenced by 35 TF genes, while MMP is regulated by 22 genes. Our analysis revealed that different key genes are modulated by distinct TFs, with 7 TFs (ZBTB26, MXD3, DRAP1, GATAD1, SSRP1, TFAP4, MXD4) simultaneously regulating both key genes. Additionally, we used miRTarBase to predicted miRNA binding sites and establish an interaction regulatory network between key genes and miRNA (**Figure 6H**). This network consists of 24 nodes and 25 edges. In this configuration, ANGPTL4 is regulated by 4 TF genes and MMP9 by 21 genes. Our results indicate that different miRNAs regulate distinct key genes, with hsa-mir-29b-3p concurrently regulating both key genes.

### Screening of candidate compounds

Our research has identified 10 candidate compounds that interact with ANGPTL4 and MMP9, sourced from DGIdb (**Supplementary Table 4**). Additionally, we have identified 10 potential therapeutic drugs for T2D combined with OA from relevant literature references (33–37). Using AutoDock, we have calculated the binding energies between the key target proteins MMP9 (PDB ID: 6ESM) and ANGPTL4 (PDB ID: 6U1U) and the candidate compounds. Upon comparing the binding energies to those of the original ligands, we have observed that in the MMP9 molecular docking group, two compounds exhibited binding energies equal to or greater than the original ligand of the protein. The drug with the highest binding affinity in this group was identified as Raloxifene (**Table 2**). In the ANGPTL4 molecular docking group, 17 compounds had binding energies equal to or greater than that of the original protein-ligand. The candidate drug showing the greatest binding activity in this group was Ezetimibe (**Table 3**). Notably, Raloxifene and S-3304 exhibited binding energies surpassing the original protein ligands of ANGPTL4 and MMP9. After analyzing the docking results with the best binding properties for MMP9 and ANGPTL4 through the Protein-Ligand Interaction Profiler website, we used PyMOL to visualize the molecular docking interactions (**Figures 6I–L**).

**Table 2:** Summary of molecular docking affinity of MMP9 with ligands.

NO.	Ligand_name	Affinity(Kcal/mol)
1	Raloxifene	-10.7
2	S-3304	-10.1
3	Ezetimibe	-9.9
4	Warfarin	-9.5
5	Curcumin Pyrazole	-9.3
6	Celecoxib	-9.3
7	Pioglitazone	-9.2
8	Curcumin	-8.8
9	Demethylwedelolactone	-8.7
10	Rosiglitazone	-8.7
11	Atorvastatin	-8.2
12	Rosuvastatin	-8.1
13	Prinomastat	-8.1
14	Incyclinide	-8.1
15	Tamsulosin	-7.8
16	Lovastatin	-7.8
17	Bevacizumab	-7.7
18	Marimastat	-6.4
19	Carboxylated Glucosamine	-6.4
20	Metformin	-6.1

The binding affinity between MMP9 and the original ligand is -10.1 Kcal/mol.

**Table 3:** Molecular docking affinity of ANGPTL4 with ligands.

NO.	Ligand_name	Affinity(Kcal/mol)
1	Ezetimibe	-8.8
2	Raloxifene	-8.6
3	Curcumin Pyrazole	-8.1
4	Pioglitazone	-8
5	Rosiglitazone	-7.9
6	S-3304	-7.8
7	Celecoxib	-7.6
8	Curcumin	-7.6
9	Warfarin	-7.3
10	Demethylwedelolactone	-7.3
11	Prinomastat	-7.1
12	Bevacizumab	-6.9
13	Atorvastatin	-6.9
14	Incyclinide	-6.5
15	Tamsulosin	-6.4
16	Rosuvastatin	-6.4
17	Carboxylated Glucosamine	-6.3
18	Marimastat	-6
19	Lovastatin	-6
20	Metformin	-5.1

The binding affinity between ANGPTL4 and the original ligand is -6.3 Kcal/mol.

## Discussion

The accumulating evidence indicates that T2D and OA has shown a strong correlation and bidirectional link (37–42). Research has identified similarities in decreased cell function, mitochondrial dysfunction, lipid metabolism disorders, chronic inflammation, and the role of pro-inflammatory cytokines like IL-1β and TNFα (43, 44). However, the intricate molecular mechanisms underlying the interplay between T2D and OA are still not fully understood. Recently, some common molecular targets of the two diseases have been identified through the bioinformatics method of weighted gene co-expression network analysis (WGCNA), including EPHA3, CEBPB, UBAP1, FZD7, IRAK3 and KDELR3, etc. Which may theoretically affect the occurrence of the two diseases by regulating signal transduction and protein activity. It has certain theoretical significance for guiding future research (45, 46). However, the identified molecular targets have not been validated by cell experiments, animal experiments, or clinical case organizations. Since T2D and OA are not the result of a single gene, many genes may play a role in both. Therefore, the bioinformatics method of PPI was used to further identify the common molecular targets in this study.

The analysis of DEGs that are common to both T2D and OA provides valuable insights into their shared pathogenesis of these two diseases. In this study, we performed GO enrichment analysis on DEGs, such as ECM. The ECM plays a crucial role in adipocyte metabolic dysfunction, which is a key factor in the onset of T2D (47). An imbalance in ECM synthesis and degradation can lead to articular cartilage destruction, which is a hallmark of OA onset (48). KEGG enrichment analysis highlighted significant pathways, such as PI3K-Akt and Wnt signaling, which plays a crucial roles in the pathogenesis of both T2D and OA. In fact, inhibiting the PI3K-Akt pathway has been shown to alleviate articular cartilage degeneration in OA (49), while activating this pathway has been found to improve insulin sensitivity, making it beneficial in the management of T2D (50). These findings highlight the shared functional enrichments and signaling pathways in T2D and OA, providing a direction for further studies on their common pathomechanisms.

To further elucidate the pathogenic molecular mechanisms between T2D and OA, we constructed a PPI analysis on shared DEGs. There are various bioinformatics analysis tools used to identify key genes. PPI is composed of proteins interacting with each other. It can systematically analyze the interaction of a large number of proteins in biological systems, explore the reaction mechanism of biological signals and material metabolism in special physiological states. It also helps to study the molecular mechanism of diseases and discovers new drug targets in the field of biomedicine. WGCNA can resolve the associations between gene collections and sample phenotypes, mapping regulatory networks between genes in gene collections, and identifying key regulatory genes. The two methods above have their own advantages. The accuracy of identifying key genes may be improved with the combination of the two methods in the future molecular target screening.

ANGPTL4 and MMP9 were identified as common critical genes. ANGPTL4 is a multifaceted 50-kDa secretory protein with a unique ~15 kD N-terminal coiled-coil domain (nANGPTL4) and a ~35 kD C-terminal fibrinogen-like domain (cANGPTL4) (51). It is predominantly expressed in metabolic tissues and is primarily produced in adipose and liver tissues, playing an essential role in lipid and glucose metabolism (52). ANGPTL4 regulates numerous cellular and physiological functions, making it a potential therapeutic target for various diseases (53). Genetic studies have linked mutations in the ANGPTL4 (E40K) gene to reduced plasma triacylglycerol and glucose levels (54, 55). Furthermore, ANGPTL4 expression levels are positively correlated with an increased risk of T2D and obesity-related diabetic phenotypes. Research by Abhishek K using a hepatocyte-specific ANGPTL4 mutant mouse model demonstrated that suppressing ANGPTL4 could prevent diet-induced obesity, reduce ectopic lipid accumulation, and enhance insulin sensitivity and glucose tolerance (56). These findings support the role of ANGPTL4 in regulating glucose homeostasis and its potential impact on T2D. Moreover, ANGPTL4 is linked to musculoskeletal diseases, including OA, and influences processes such as bone resorption, cartilage degradation, angiogenesis, and vascular permeability (57, 58). It also participates in receptor-mediated and intracellular activities, including NF-κB-regulated inflammatory responses and interaction with sirtuin1 (59). A recent study found that silencing ANGPTL4 in animal models alleviated OA progression, inhibited the sirtuin1/NF-κB signaling pathway, and reduced TNFα-induced chondrocyte inflammation and apoptosis (60). Collectively, these studies highlight the significant impact of ANGPTL4 on the development of T2D and OA. Our clinical sample analysis confirmed ANGPTL4 expression in the pathological tissues of patients with T2D + OA.

The matrix metalloproteinases family, consisting of multiple members, primarily regulates various cellular behaviors such as proliferation, migration, differentiation, apoptosis, and host defense (61). Among these members, MMP9, a type IX collagenase, is prevalent in non-infected normal connective tissues, where it aids in the degradation of the extracellular matrix and the inflammatory response (62). Research has shown a link between OA and both local and systemic low-grade inflammation, with MMP9 playing a pivotal role (61, 63). In inflammatory conditions, MMPs are produced by body tissues, contributing to the degradation of the cartilage extracellular matrix, which is primarily compose of proteoglycans and collagen. MMP9, in particular, targets type IX collagen, accelerating extracellular matrix degradation and thereby establishing a direct correlation with the pathogenesis of OA (64).

Furthermore, MMP9 has been implicated in the progression of diabetic osteoarthritis. In an animal study on diabetic OA, MMP9 overexpression was observed in rat cartilage, leading to inhibition of the anti-apoptotic protein (B-cell lymphoma-2) and an increase in apoptotic cartilage cells (65). Moreover, MMP9 overexpression suppressed the expression of cartilage markers collagen type II alpha 1 (COL2a1) and collagen type I alpha 1 (COL1a1), while its inhibition reversed the decrease in COL2a1 and COL1a1expression. An previous study also demonstrated that plasma MMP9 level was significantly elevated in early OA patients and positively correlated with the severity of clinical symptoms (total Lequesne’s scores), even when imaging features did not indicate articular cartilage degeneration (66). This suggests that MMP9 could potentially serve as an early diagnostic marker of OA. These findings, which are consistent with our study, highlight the high expression of MMP9 in both the pathological and cartilage tissues of T2D and OA patients. This underscores the regulatory mechanism of ANGPTL4 and MMP9 in T2D + OA. As discovered in this study, the expression of MMP9 and ANGPTL4 has a positive correlation between blood glucose and KL in patients, which suggests a causal association between MMP9, ANGPTL4 with T2D and OA. Therefore, ANGPTL4 and MMP9 may have potential value in diagnosing and treating both diseases. However, T2D and OA are not the result of a single gene, and many genes or environmental factors may play a role in both (67). Arruda found evidences of colocalization at 18 genomic loci to T2D and OA, and these findings support enrichment for lipid metabolism and skeletal formation pathways for signals underpinning T2D comorbidities with OA (68). At the current, most study don’t have sufficient data on phenotype, other potential predictors, clinical and imaging parameters to fully elucidate causal relationships between genes and diseases. The more detailed study of the queue population is needed.

Our investigation focused on understanding the relationship between miRNAs, TFs, and the expression of ANGPTL4 and MMP9. Previous research indicates that the miR-29 family, including miR-29a, miR-29b, and miR-29c, plays a significantly role in the pathogenesis of T2D and OA (69–71). Le found increased expression of hsa-miR-29b-3p in both OA mouse model experiments and clinical OA tissue samples, indicating its role in early OA progression (72). Furthermore, hsa-miR-29b-3p has been linked to the NF-κB and WNT signaling pathways and is implicated in the onset of diabetes. Marttila noted that hsa-miR-29b-3p’s predicted targets are enriched in the insulin signaling pathway, with a positive correlation between its overall expression and serum VLDL lipid and triglyceride levels, potentially influence glucose tolerance (70). Animal models have also demonstrated that hsa-miR-29b-3p’s contributes to insulin resistance and can inhibit insulin-stimulated glucose uptake, affecting blood glucose levels (73, 74). Our findings suggest that seven TFs–ZBTB26, MXD3, DRAP1, GATAD1, SSRP1, TFAP4, and MXD4 concurrently regulate ANGPTL4 and MMP9, potentially influencing the development of T2D and OA. However, there is currently limited evidence confirming their involvement in these diseases. Further research into these miRNAs and TFs may provide a valuable insight into the mechanisms of T2D and OA and could potentially lead to new therapeutic targets.

Developing accurate predictive models for diagnosing diseases and assessing their severity is crucial for risk stratification, tailoring treatments, and improving patient quality of life. However, there is a notable gap in predictive models for the early detection of OA induced by T2D. To address this gap, we developed a diagnostic prediction model using external data for ANGPTL4 and MMP9. Our model demonstrated high accuracy, suggesting that ANGPTL4 and MMP9 could serve as preventive and diagnostic biomarkers for T2D and OA, providing a theoretical foundation for understanding the molecular mechanisms underlying their co-occurrence.

The molecular docking insights from our study suggest that Raloxifene, Ezetimibe, and S-3304 may have unique therapeutic potential for T2D and OA. Previous studies have shown that raloxifene, a selective estrogen receptor modulator, can prevent diabetes onset and improve bone material properties in diabetes-prone rats (75). Laura Tinti found that raloxifene had chondroprotective effects in human osteoarthritis chondrocytes (76). S-3304, a novel D-tryptophan derivative, is an MMP inhibitor that has been shown to reduce extracellular matrix degradation and inhibit angiogenesis, tumor growth, invasion, and metastasis, primarily in cancer research (77). Ezetimibe, a cholesterol absorption inhibitor, has been found to inhibit the NF-κB pathway, attenuate IL-1β-induced extracellular matrix degradation, and reduce MMP expression levels induced by IL-1β, thereby exerting a protective effect (78). These findings support further experimental and clinical research in this area.

Despite the contributions of our study, it is important to acknowledge its limitations. Firstly, due to the distinct nature of the two diseases, it was challenging to find a suitable dataset with identical sample tissue in public databases. The limited clinical information in these databases poses a risk of sample contamination, potentially leading to biased analytical results. Secondly, due to the small sample size of clinical patients included, there may be biases in the correlation between MMP9, ANGPTL4 and body weight, blood glucose, bone mineral density, Kellgren Lawrence grade, GHbA1c. Thirdly, due to limitations in our laboratory conditions and project funding, we are currently unable to further validate the efficacy and safety of the identified potential drugs *in vitro*.

## Conclusion

Our study sheds light on shared signaling pathways, biomarkers, potential therapeutic agents, and diagnostic models for T2D and OA. These findings offer novel insights into the pathogenesis, diagnosis, and treatment approaches for T2D combined with OA, paving the way for future research.

## Data availability statement

The original contributions presented in the study are included in the article/**Supplementary Material**. Further inquiries can be directed to the corresponding authors.

## Ethics statement

The studies involving humans were approved by the Institutional Review Board of Panzhihua Central Hospital. The studies were conducted in accordance with the local legislation and institutional requirements. The participants provided their written informed consent to participate in this study.

## Author contributions

GM: Writing – review & editing, Writing – original draft, Validation, Project administration, Methodology, Investigation, Formal Analysis, Data curation. WX: Writing – review & editing, Project administration, Methodology. LW: Writing – original draft, Validation, Methodology, Formal Analysis, Data curation. HW: Writing – original draft, Methodology, Investigation, Data curation. SX: Writing – original draft, Methodology. LZ: Writing – review & editing. SL: Writing – review & editing. JZ: Writing – original draft. ZL: Writing – original draft. YL: Writing – review & editing, Supervision, Project administration, Methodology. JL: Writing – review & editing, Supervision, Project administration, Methodology.
