# Supplementary figures and images for: Unveiling the bioinformatic genes and their involved regulatory mechanisms in type 2 diabetes combined with osteoarthritis
Source: Front Immunol. 2024 Aug 8;15:1353915. doi: 10.3389/fimmu.2024.1353915 (PMC11338775; doi:10.3389/fimmu.2024.1353915)

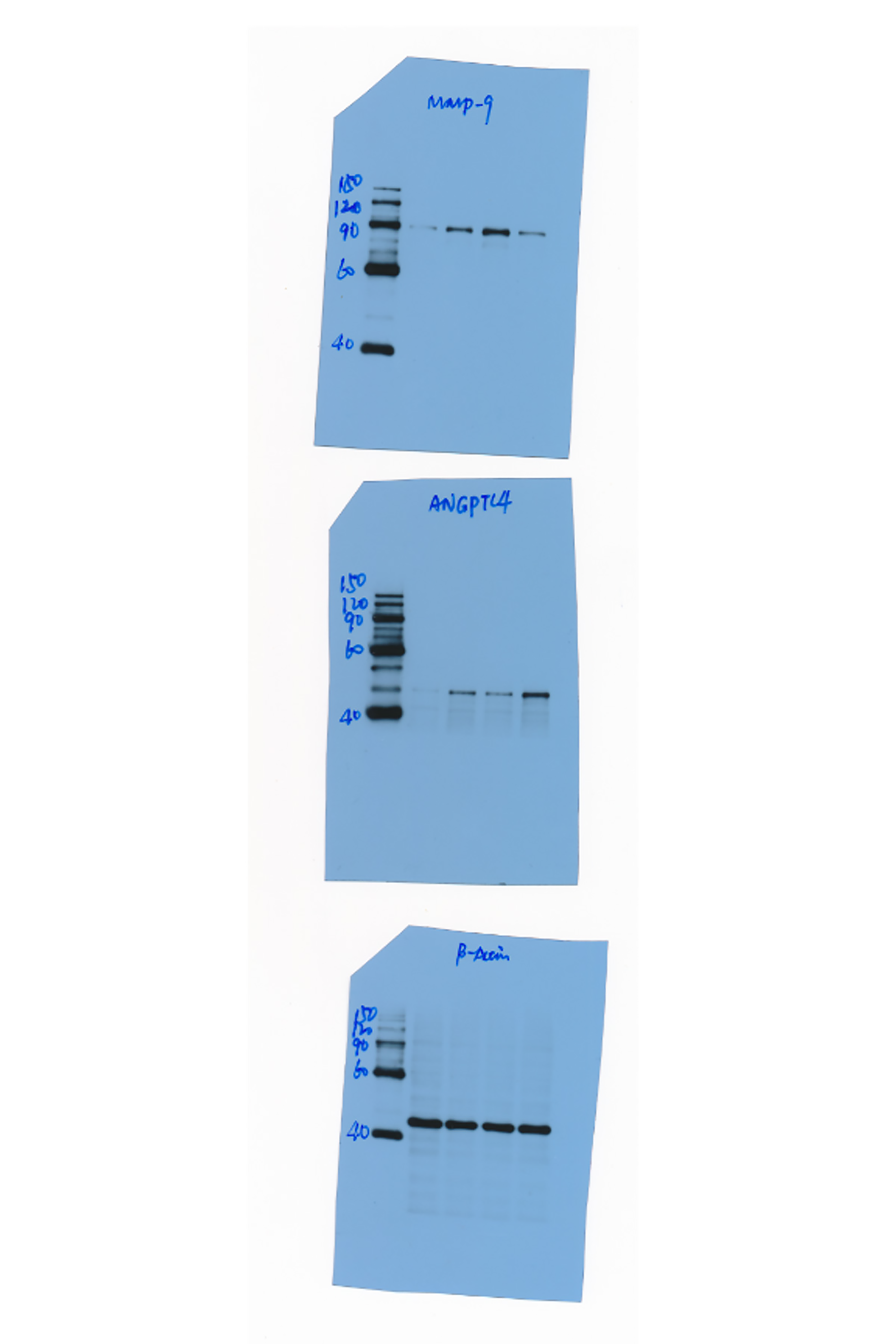

Supplement: Supplementary file 1 [file Image_1.tif]
