# Supplementary material for: Unveiling the bioinformatic genes and their involved regulatory mechanisms in type 2 diabetes combined with osteoarthritis
Source: Front Immunol. 2024 Aug 8;15:1353915. doi: 10.3389/fimmu.2024.1353915 (PMC11338775; doi:10.3389/fimmu.2024.1353915)
Supplement: Supplementary file 2 [file Table_1.docx]

| Inclusion criteria | | Exclusion criteria |
| --- | --- | --- |
| T2D group | The enrolled population met the T2D diagnostic criteria, but they were not diagnosed with OA and had no related complications | 1.Bone diseases (such as osteoporosis, rheumatoid osteoarthritis, rheumatoid osteoarthritis, gouty joints)  Inflammation) and the clear history of bone surgery or trauma.  2.Other metabolic diseases, such as hypocalcemia, hypercalcemia, hyperlipidemia, hypolipemia, hyperuricemia, thyroid function disease.  3.Serious diseases: such as cerebral infarction, cerebral hemorrhage, serious coronary heart disease, kidney disease, cancer, severe infection, etc.  4.The history of surgery or trauma in the past 6 months.  5.The history of glucocorticoid drugs,immunosuppressants, angiogenesis inhibitors, thiazide diuretics,  antipsychotic drugs, beta blockers in the past 6 months.  6.Over 75 years old or under 18 years old.  7.Pregnant women. |
| OA group | The enrolled population  met the diagnostic  criteria for OA, but they  were not diagnosed with  T2D and had no related  Complications |  |
| T2D combined with OA group | The enrolled population met the diagnostic criteria for T2D and OA, but they had no other  Complications |  |
| Control group | healthy adults |  |

**Table1 The inclusion and exclusion criteria for the clinical study**

T2D, Type 2 Diabetes Mellitus. OA, Osteoarthritis
