# Supplementary material for: Unveiling the bioinformatic genes and their involved regulatory mechanisms in type 2 diabetes combined with osteoarthritis
Source: Front Immunol. 2024 Aug 8;15:1353915. doi: 10.3389/fimmu.2024.1353915 (PMC11338775; doi:10.3389/fimmu.2024.1353915)
Supplement: Supplementary file 3 [file Table_2.docx]

| Primer Name | Primer Information | Base sequence (5`-3`) | | Tm | CG% | Product length (bp) |
| --- | --- | --- | --- | --- | --- | --- |
| H-ACTIN | NM_001101 | sense | GTCCACCGCAAATGCTTCTA | 58.7 | 50 | 190 |
|  |  | antisense | TGCTGTCACCTTCACCGTTC | 58.9 | 55 |  |
| H-MMP9 | NM_004994.2 | sense | TCGAACTTTGACAGCGACAAG | 58.9 | 47.6 | 152 |
|  |  | antisense | TCAGTGAAGCGGTACATAGGGT | 59 | 50 |  |
| HANGPTL4 | NM_139314 | sense | TGGACCACAAGCACCTAGACC | 61.7 | 57.1 | 221 |
|  |  | antisense | TGAGGTCATCTTGCAGTTCACC | 60.6 | 50 |  |

**Table1 Primers for qRT-PCR**
