# Supplementary material for: Unveiling the bioinformatic genes and their involved regulatory mechanisms in type 2 diabetes combined with osteoarthritis
Source: Front Immunol. 2024 Aug 8;15:1353915. doi: 10.3389/fimmu.2024.1353915 (PMC11338775; doi:10.3389/fimmu.2024.1353915)
Supplement: Supplementary file 4 [file Table_3.docx]

| samples | gender | age（years） | IBM(kg/m²) | fasting glucose(mmol/l) | GHbA1c(%) | Kellgren-Lawrence grade | Bone mass grade | MMP9 expression levels | ANGPLT4 expression levels |
| --- | --- | --- | --- | --- | --- | --- | --- | --- | --- |
| Normal control1 | male | 64 | 22.83 | 5.14 | 3.45 | 0 | 1 | 0.39 | 0.19 |
| Normal control2 | male | 51 | 25.53 | 5.30 | 5.06 | 0 | 1 | 0.03 | 0.47 |
| Normal control3 | female | 59 | 23.14 | 4.08 | 3.97 | 0 | 1 | 0.21 | 1.41 |
| Normal control4 | female | 61 | 25.48 | 5.67 | 3.77 | 0 | 2 | 0.29 | 0.50 |
| Normal control5 | female | 55 | 20.96 | 5.49 | 3.07 | 0 | 1 | 0.21 | 0.13 |
| Normal control6 | male | 65 | 28.94 | 5.42 | 5.59 | 1 | 1 | 1.47 | 0.12 |
| Normal control7 | male | 60 | 26.44 | 5.82 | 5.78 | 0 | 1 | 0.48 | 0.37 |
| Normal control8 | female | 62 | 28.04 | 4.89 | 5.52 | 0 | 1 | 0.67 | 0.19 |
| Normal control9 | female | 54 | 22.53 | 4.87 | 3.72 | 0 | 1 | 0.72 | 0.35 |
| T2D1 | male | 69 | 23.21 | 8.38 | 6.83 | 0 | 2 | 1.00 | 1.00 |
| T2D2 | male | 55 | 29.42 | 9.24 | 7.94 | 1 | 1 | 2.20 | 3.11 |
| T2D3 | female | 52 | 24.19 | 7.52 | 5.98 | 0 | 1 | 0.69 | 1.79 |
| T2D4 | female | 48 | 23.58 | 14.28 | 8.45 | 2 | 1 | 3.77 | 2.40 |
| T2D5 | female | 57 | 27.27 | 12.02 | 7.94 | 1 | 1 | 2.01 | 2.58 |
| T2D6 | male | 68 | 26.02 | 7.23 | 5.30 | 1 | 1 | 1.59 | 2.00 |
| T2D7 | male | 55 | 25.65 | 8.02 | 6.05 | 0 | 1 | 0.61 | 0.93 |
| T2D8 | male | 57 | 21.06 | 8.29 | 7.02 | 1 | 1 | 1.39 | 1.06 |
| T2D+OA1 | male | 62 | 26.80 | 15.54 | 12.21 | 4 | 1 | 2.30 | 0.45 |
| T2D+OA2 | female | 50 | 27.23 | 11.72 | 11.21 | 4 | 1 | 1.73 | 0.73 |
| T2D+OA3 | male | 64 | 23.11 | 14.99 | 9.59 | 3 | 1 | 0.77 | 1.13 |
| T2D+OA4 | female | 53 | 22.78 | 11.10 | 12.22 | 4 | 1 | 2.87 | 3.53 |
| OA1 | male | 58 | 24.32 | 4.61 | 3.86 | 3 | 1 | 0.13 | 0.24 |
| OA2 | female | 65 | 28.66 | 6.78 | 7.02 | 4 | 1 | 5.79 | 2.00 |
| OA3 | male | 61 | 31.69 | 6.69 | 6.84 | 4 | 1 | 2.05 | 1.89 |
| OA4 | male | 70 | 25.71 | 6.72 | 6.25 | 4 | 2 | 2.28 | 2.29 |
| OA5 | female | 56 | 29.31 | 6.24 | 7.21 | 4 | 1 | 2.38 | 1.84 |
| OA6 | female | 64 | 28.30 | 5.69 | 5.61 | 4 | 1 | 1.17 | 2.09 |
| OA7 | male | 68 | 26.03 | 4.09 | 5.80 | 3 | 2 | 1.08 | 0.33 |

**Kellgren-Lawrence grade:0-** no osteoarthritic changes. 1- Doubtful narrowing of joint space and possible osteophytic lipping. 2- definite osteophytes and possible narrowing of joint space. 3- moderate multiple osteophytes, definite narrowing of joint space, some sclerosis, and possible deformity of bone ends. 4- large osteophytes, marked narrowing of joint space, severe sclerosis and definite deformity of bone ends

**Bone mass density grade:**1- A value for BMD within 1.0 SD of the young adult female reference mean (T-score greater than or equal to -1.0 SD). 2- A value for BMD more than 1.0 but less than 2.5 SD below the young adult female reference mean (T-score less than -1 and greater than -2.5 SD). 3- A value for BMD 2.5 or more SD below the young adult female reference mean (T-score less than or equal to -2.5 SD). 4-A value for BMD more than 2.5 SD below the young adult female reference mean in the presence of one or more fragility fractures.
