# Supplementary material for: Unveiling the bioinformatic genes and their involved regulatory mechanisms in type 2 diabetes combined with osteoarthritis
Source: Front Immunol. 2024 Aug 8;15:1353915. doi: 10.3389/fimmu.2024.1353915 (PMC11338775; doi:10.3389/fimmu.2024.1353915)
Supplement: Supplementary file 5 [file Table_4.docx]

| Candidate drug | Source of candidate drug | PubChem CID of candidate drug |
| --- | --- | --- |
| Celecoxib | DGIdb | 2662 |
| Marimastat | DGIdb | 119031 |
| Demethylwedelolactone | DGIdb | 5489605 |
| Curcumin | DGIdb | 969516 |
| Prinomastat | DGIdb | 466151 |
| Tozuleristide | DGIdb | 121488172 |
| Bevacizumab | DGIdb | 24801581 |
| S-3304 | DGIdb | 10718956 |
| Incyclinide | DGIdb | 54678924 |
| Curcumin Pyrazole | DGIdb | 135494223 |
| Carboxylated Glucosamine | DGIdb | 44411803 |
| Metformin | literature | 4091 |
| Pioglitazone | literature | 4829 |
| Rosiglitazone | literature | 77999 |
| Atorvastatin | literature | 60823 |
| Rosuvastatin | literature | 446157 |
| Lovastatin | literature | 53232 |
| Tamsulosin | literature | 129211 |
| Warfarin | literature | [54678486](https://pubchem.ncbi.nlm.nih.gov/compound/54678486) |
| Raloxifene | literature | 5035 |
| Ezetimibe | literature | 150311 |

**Table 3 Candidate Drug**
